# Supplementary material for: Multi‐dataset identification of innovative feature genes and molecular mechanisms in keratoconus
Source: J Cell Mol Med. 2024 Sep 19;28(18):e70079. doi: 10.1111/jcmm.70079 (PMC11412914; doi:10.1111/jcmm.70079)
Supplement: Supplementary file 2 — Material S2. [file JCMM-28-e70079-s002.zip › 7.GSEA analysis/1.ARL11/KEGG_CYTOKINE_CYTOKINE_RECEPTOR_INTERACTION.html]

Details for gene set KEGG\_CYTOKINE\_CYTOKINE\_RECEPTOR\_INTERACTION[GSEA]

|  || Dataset | ARL11.group.cls#h\_versus\_l |
| Phenotype | group.cls#h\_versus\_l |
| Upregulated in class | l |
| GeneSet | KEGG\_CYTOKINE\_CYTOKINE\_RECEPTOR\_INTERACTION |
| Enrichment Score (ES) | -0.33560786 |
| Normalized Enrichment Score (NES) | -1.0060666 |
| Nominal p-value | 0.45825243 |
| FDR q-value | 1.0 |
| FWER p-Value | 1.0 |
Table: GSEA Results Summary

  

Fig 1: Enrichment plot: KEGG\_CYTOKINE\_CYTOKINE\_RECEPTOR\_INTERACTION      
 Profile of the Running ES Score & Positions of GeneSet Members on the Rank Ordered List

  

| SYMBOL | TITLE | RANK IN GENE LIST | RANK METRIC SCORE | RUNNING ES | CORE ENRICHMENT || 1 | IL7R | na | 136 | 0.619 | 0.0120 | No |
| 2 | TNFSF14 | na | 242 | 0.560 | 0.0234 | No |
| 3 | IL21 | na | 309 | 0.534 | 0.0352 | No |
| 4 | CCL4L2 | na | 828 | 0.413 | 0.0308 | No |
| 5 | LEP | na | 970 | 0.392 | 0.0369 | No |
| 6 | IL3RA | na | 1221 | 0.364 | 0.0390 | No |
| 7 | CCL23 | na | 1393 | 0.347 | 0.0430 | No |
| 8 | IFNL3 | na | 1401 | 0.346 | 0.0517 | No |
| 9 | IL26 | na | 1410 | 0.345 | 0.0604 | No |
| 10 | TNFRSF25 | na | 1762 | 0.314 | 0.0583 | No |
| 11 | CXCR2 | na | 1957 | 0.299 | 0.0604 | No |
| 12 | TGFBR2 | na | 2250 | 0.279 | 0.0591 | No |
| 13 | LTA | na | 2268 | 0.278 | 0.0658 | No |
| 14 | TNFRSF19 | na | 2279 | 0.278 | 0.0727 | No |
| 15 | BMPR1B | na | 2280 | 0.278 | 0.0798 | No |
| 16 | XCL1 | na | 2366 | 0.272 | 0.0844 | No |
| 17 | CCL26 | na | 2398 | 0.270 | 0.0904 | No |
| 18 | CSF1R | na | 2453 | 0.268 | 0.0958 | No |
| 19 | IL2RB | na | 2535 | 0.263 | 0.1002 | No |
| 20 | PPBP | na | 2983 | 0.241 | 0.0934 | No |
| 21 | IL4R | na | 3015 | 0.240 | 0.0987 | No |
| 22 | TNFSF4 | na | 3031 | 0.239 | 0.1044 | No |
| 23 | NGFR | na | 3348 | 0.225 | 0.1011 | No |
| 24 | IFNA6 | na | 3473 | 0.220 | 0.1032 | No |
| 25 | IL3 | na | 3483 | 0.220 | 0.1086 | No |
| 26 | CXCL13 | na | 3555 | 0.217 | 0.1121 | No |
| 27 | OSM | na | 3598 | 0.215 | 0.1164 | No |
| 28 | IL17RB | na | 3615 | 0.215 | 0.1215 | No |
| 29 | IL7 | na | 3809 | 0.208 | 0.1212 | No |
| 30 | CXCL6 | na | 4243 | 0.192 | 0.1136 | No |
| 31 | IFNA14 | na | 4269 | 0.191 | 0.1178 | No |
| 32 | IL19 | na | 4416 | 0.186 | 0.1184 | No |
| 33 | CCR10 | na | 4569 | 0.180 | 0.1186 | No |
| 34 | CCL5 | na | 4596 | 0.179 | 0.1225 | No |
| 35 | IFNA16 | na | 4914 | 0.169 | 0.1176 | No |
| 36 | TNFRSF10C | na | 5214 | 0.160 | 0.1131 | No |
| 37 | ACVR2B | na | 5771 | 0.143 | 0.1006 | No |
| 38 | HGF | na | 5944 | 0.138 | 0.0992 | No |
| 39 | PDGFRA | na | 5971 | 0.138 | 0.1020 | No |
| 40 | IFNA4 | na | 6226 | 0.131 | 0.0980 | No |
| 41 | CCR5 | na | 6412 | 0.126 | 0.0959 | No |
| 42 | IL2RA | na | 6518 | 0.124 | 0.0960 | No |
| 43 | IL6 | na | 6613 | 0.121 | 0.0964 | No |
| 44 | EDA2R | na | 6741 | 0.118 | 0.0958 | No |
| 45 | IL18R1 | na | 6788 | 0.117 | 0.0974 | No |
| 46 | CXCR4 | na | 7115 | 0.109 | 0.0908 | No |
| 47 | PDGFA | na | 7165 | 0.108 | 0.0922 | No |
| 48 | CCR6 | na | 7305 | 0.105 | 0.0909 | No |
| 49 | ACVR1B | na | 7347 | 0.105 | 0.0924 | No |
| 50 | FLT3 | na | 7449 | 0.103 | 0.0921 | No |
| 51 | TNFSF9 | na | 7498 | 0.102 | 0.0933 | No |
| 52 | CX3CR1 | na | 7560 | 0.101 | 0.0941 | No |
| 53 | TGFB2 | na | 7601 | 0.100 | 0.0955 | No |
| 54 | EPO | na | 7618 | 0.099 | 0.0976 | No |
| 55 | FLT3LG | na | 7704 | 0.098 | 0.0977 | No |
| 56 | IFNE | na | 7843 | 0.096 | 0.0962 | No |
| 57 | PLEKHO2 | na | 8124 | 0.090 | 0.0904 | No |
| 58 | BMPR2 | na | 8199 | 0.088 | 0.0905 | No |
| 59 | CXCR6 | na | 8660 | 0.080 | 0.0792 | No |
| 60 | GHR | na | 8677 | 0.080 | 0.0808 | No |
| 61 | IL23R | na | 8686 | 0.080 | 0.0826 | No |
| 62 | CCL3 | na | 8722 | 0.079 | 0.0836 | No |
| 63 | VEGFC | na | 8842 | 0.077 | 0.0822 | No |
| 64 | IL17B | na | 8854 | 0.077 | 0.0838 | No |
| 65 | AMHR2 | na | 8935 | 0.076 | 0.0835 | No |
| 66 | IL20RA | na | 9017 | 0.075 | 0.0830 | No |
| 67 | TNFRSF14 | na | 9164 | 0.072 | 0.0807 | No |
| 68 | PF4 | na | 9945 | 0.061 | 0.0596 | No |
| 69 | IL13 | na | 10076 | 0.059 | 0.0574 | No |
| 70 | TNFSF15 | na | 10321 | 0.056 | 0.0517 | No |
| 71 | CNTF | na | 10488 | 0.054 | 0.0483 | No |
| 72 | IL15RA | na | 10561 | 0.053 | 0.0476 | No |
| 73 | TNFSF10 | na | 10966 | 0.048 | 0.0371 | No |
| 74 | IL1RAP | na | 11097 | 0.047 | 0.0345 | No |
| 75 | EGFR | na | 11115 | 0.046 | 0.0352 | No |
| 76 | KITLG | na | 11152 | 0.046 | 0.0354 | No |
| 77 | IL13RA1 | na | 11669 | 0.040 | 0.0214 | No |
| 78 | TNFSF13B | na | 11811 | 0.038 | 0.0183 | No |
| 79 | CXCL2 | na | 11903 | 0.037 | 0.0166 | No |
| 80 | IL20RB | na | 11926 | 0.037 | 0.0169 | No |
| 81 | CXCL1 | na | 12091 | 0.035 | 0.0131 | No |
| 82 | IFNL2 | na | 12511 | 0.031 | 0.0017 | No |
| 83 | TSLP | na | 12541 | 0.031 | 0.0017 | No |
| 84 | CCR3 | na | 12826 | 0.028 | -0.0058 | No |
| 85 | IL12A | na | 13401 | 0.024 | -0.0218 | No |
| 86 | CCL1 | na | 13420 | 0.024 | -0.0218 | No |
| 87 | CCL11 | na | 13583 | 0.022 | -0.0259 | No |
| 88 | EGF | na | 13590 | 0.022 | -0.0255 | No |
| 89 | FLT1 | na | 13684 | 0.021 | -0.0276 | No |
| 90 | EPOR | na | 13761 | 0.021 | -0.0293 | No |
| 91 | TNFSF12 | na | 13786 | 0.021 | -0.0295 | No |
| 92 | CX3CL1 | na | 13861 | 0.020 | -0.0311 | No |
| 93 | TNFRSF10A | na | 13975 | 0.019 | -0.0339 | No |
| 94 | VEGFB | na | 14052 | 0.018 | -0.0356 | No |
| 95 | CTF1 | na | 14511 | 0.015 | -0.0485 | No |
| 96 | IL15 | na | 14630 | 0.014 | -0.0516 | No |
| 97 | CXCR3 | na | 14830 | 0.012 | -0.0570 | No |
| 98 | IFNA2 | na | 15087 | 0.011 | -0.0642 | No |
| 99 | TNFRSF10B | na | 15315 | 0.009 | -0.0705 | No |
| 100 | KDR | na | 15337 | 0.009 | -0.0709 | No |
| 101 | CSF1 | na | 15339 | 0.009 | -0.0707 | No |
| 102 | IL11RA | na | 15527 | 0.007 | -0.0760 | No |
| 103 | IL17RA | na | 15710 | 0.006 | -0.0811 | No |
| 104 | IL22 | na | 15849 | 0.005 | -0.0850 | No |
| 105 | IFNAR1 | na | 15880 | 0.005 | -0.0857 | No |
| 106 | CCL18 | na | 15885 | 0.005 | -0.0857 | No |
| 107 | FASLG | na | 15958 | 0.004 | -0.0877 | No |
| 108 | KIT | na | 16102 | 0.003 | -0.0918 | No |
| 109 | BMPR1A | na | 16675 | -0.001 | -0.1084 | No |
| 110 | IL5RA | na | 16947 | -0.003 | -0.1161 | No |
| 111 | CXCL5 | na | 16970 | -0.003 | -0.1167 | No |
| 112 | GH2 | na | 17060 | -0.004 | -0.1192 | No |
| 113 | CCL24 | na | 17148 | -0.004 | -0.1216 | No |
| 114 | CXCL14 | na | 17774 | -0.009 | -0.1395 | No |
| 115 | CCL17 | na | 17843 | -0.009 | -0.1412 | No |
| 116 | CCR4 | na | 18343 | -0.013 | -0.1554 | No |
| 117 | TNFRSF1A | na | 18347 | -0.013 | -0.1552 | No |
| 118 | ACVR2A | na | 18737 | -0.015 | -0.1660 | No |
| 119 | PRL | na | 18902 | -0.016 | -0.1704 | No |
| 120 | CCL3L3 | na | 19075 | -0.017 | -0.1749 | No |
| 121 | IL18 | na | 19446 | -0.020 | -0.1851 | No |
| 122 | TGFBR1 | na | 19919 | -0.023 | -0.1982 | No |
| 123 | IL2RG | na | 20265 | -0.026 | -0.2076 | No |
| 124 | IL6R | na | 20441 | -0.027 | -0.2120 | No |
| 125 | IFNB1 | na | 20512 | -0.028 | -0.2133 | No |
| 126 | TNFSF13 | na | 20555 | -0.028 | -0.2138 | No |
| 127 | TNFRSF13C | na | 20657 | -0.029 | -0.2160 | No |
| 128 | CD27 | na | 20847 | -0.030 | -0.2207 | No |
| 129 | BMP7 | na | 20858 | -0.030 | -0.2202 | No |
| 130 | CNTFR | na | 21425 | -0.035 | -0.2357 | No |
| 131 | FLT4 | na | 21654 | -0.036 | -0.2414 | No |
| 132 | INHBC | na | 21714 | -0.037 | -0.2421 | No |
| 133 | XCR1 | na | 21803 | -0.038 | -0.2437 | No |
| 134 | IFNGR1 | na | 21859 | -0.038 | -0.2443 | No |
| 135 | CCL16 | na | 21900 | -0.039 | -0.2445 | No |
| 136 | CCL28 | na | 22053 | -0.040 | -0.2479 | No |
| 137 | CXCL9 | na | 22086 | -0.041 | -0.2478 | No |
| 138 | CD70 | na | 22328 | -0.043 | -0.2536 | No |
| 139 | LTBR | na | 22485 | -0.045 | -0.2570 | No |
| 140 | TNF | na | 22702 | -0.047 | -0.2621 | No |
| 141 | TNFRSF18 | na | 23017 | -0.050 | -0.2699 | No |
| 142 | RELT | na | 23573 | -0.057 | -0.2845 | No |
| 143 | TPO | na | 23798 | -0.060 | -0.2895 | No |
| 144 | EDAR | na | 23894 | -0.061 | -0.2907 | No |
| 145 | FAS | na | 24099 | -0.064 | -0.2949 | No |
| 146 | IL6ST | na | 24197 | -0.065 | -0.2961 | No |
| 147 | CXCL16 | na | 24609 | -0.071 | -0.3062 | No |
| 148 | TNFRSF11A | na | 24634 | -0.071 | -0.3050 | No |
| 149 | IL12RB1 | na | 24647 | -0.071 | -0.3035 | No |
| 150 | CSF3R | na | 24746 | -0.073 | -0.3045 | No |
| 151 | CCL15 | na | 24923 | -0.075 | -0.3077 | No |
| 152 | LIF | na | 25088 | -0.077 | -0.3104 | No |
| 153 | CCL25 | na | 25455 | -0.082 | -0.3189 | No |
| 154 | TGFB1 | na | 25459 | -0.082 | -0.3169 | No |
| 155 | CCL2 | na | 25493 | -0.083 | -0.3157 | No |
| 156 | CCL8 | na | 25621 | -0.085 | -0.3172 | No |
| 157 | IL9R | na | 25644 | -0.085 | -0.3157 | No |
| 158 | CCL19 | na | 25762 | -0.087 | -0.3169 | No |
| 159 | CCR8 | na | 26010 | -0.090 | -0.3217 | No |
| 160 | IL10RA | na | 26348 | -0.096 | -0.3290 | No |
| 161 | CCL27 | na | 26351 | -0.096 | -0.3266 | No |
| 162 | IFNAR2 | na | 26387 | -0.096 | -0.3251 | No |
| 163 | CD40 | na | 26388 | -0.096 | -0.3226 | No |
| 164 | ACVR1 | na | 26478 | -0.098 | -0.3227 | No |
| 165 | TNFSF11 | na | 26518 | -0.099 | -0.3213 | No |
| 166 | CCR2 | na | 26561 | -0.100 | -0.3199 | No |
| 167 | LEPR | na | 26569 | -0.100 | -0.3175 | No |
| 168 | TNFRSF10D | na | 26768 | -0.104 | -0.3206 | No |
| 169 | IL12B | na | 26869 | -0.106 | -0.3208 | No |
| 170 | INHBB | na | 26936 | -0.107 | -0.3199 | No |
| 171 | IL23A | na | 27477 | -0.118 | -0.3326 | Yes |
| 172 | TNFSF8 | na | 27491 | -0.119 | -0.3299 | Yes |
| 173 | TNFSF18 | na | 27504 | -0.119 | -0.3272 | Yes |
| 174 | IFNGR2 | na | 27521 | -0.119 | -0.3246 | Yes |
| 175 | MET | na | 27578 | -0.120 | -0.3231 | Yes |
| 176 | IL10RB | na | 27648 | -0.122 | -0.3219 | Yes |
| 177 | IL12RB2 | na | 27665 | -0.123 | -0.3192 | Yes |
| 178 | IL21R | na | 27687 | -0.123 | -0.3167 | Yes |
| 179 | AMH | na | 27759 | -0.125 | -0.3155 | Yes |
| 180 | PDGFC | na | 27761 | -0.125 | -0.3123 | Yes |
| 181 | TNFRSF12A | na | 27848 | -0.127 | -0.3115 | Yes |
| 182 | EDA | na | 28000 | -0.131 | -0.3126 | Yes |
| 183 | PF4V1 | na | 28323 | -0.139 | -0.3183 | Yes |
| 184 | IL1B | na | 28530 | -0.144 | -0.3206 | Yes |
| 185 | CCR1 | na | 28538 | -0.144 | -0.3170 | Yes |
| 186 | IL1A | na | 28564 | -0.145 | -0.3140 | Yes |
| 187 | CSF2RB | na | 28575 | -0.145 | -0.3106 | Yes |
| 188 | VEGFA | na | 28646 | -0.148 | -0.3088 | Yes |
| 189 | TNFRSF11B | na | 28701 | -0.149 | -0.3065 | Yes |
| 190 | TNFRSF8 | na | 28747 | -0.151 | -0.3039 | Yes |
| 191 | IL9 | na | 28748 | -0.151 | -0.3001 | Yes |
| 192 | MPL | na | 28953 | -0.155 | -0.3020 | Yes |
| 193 | IL22RA1 | na | 28959 | -0.155 | -0.2981 | Yes |
| 194 | CCL13 | na | 29231 | -0.164 | -0.3018 | Yes |
| 195 | CXCL8 | na | 29280 | -0.165 | -0.2989 | Yes |
| 196 | TNFRSF21 | na | 29388 | -0.169 | -0.2977 | Yes |
| 197 | CLCF1 | na | 29418 | -0.170 | -0.2941 | Yes |
| 198 | LIFR | na | 29466 | -0.171 | -0.2911 | Yes |
| 199 | PDGFRB | na | 29523 | -0.173 | -0.2882 | Yes |
| 200 | TNFRSF17 | na | 29554 | -0.174 | -0.2846 | Yes |
| 201 | GH1 | na | 29711 | -0.179 | -0.2845 | Yes |
| 202 | CSF3 | na | 29731 | -0.180 | -0.2805 | Yes |
| 203 | CCR7 | na | 29761 | -0.180 | -0.2767 | Yes |
| 204 | CSF2 | na | 30130 | -0.191 | -0.2824 | Yes |
| 205 | CXCL12 | na | 30202 | -0.194 | -0.2794 | Yes |
| 206 | IL1R1 | na | 30266 | -0.196 | -0.2762 | Yes |
| 207 | CXCL3 | na | 30346 | -0.199 | -0.2734 | Yes |
| 208 | IL1R2 | na | 30605 | -0.208 | -0.2755 | Yes |
| 209 | BMP2 | na | 30615 | -0.208 | -0.2704 | Yes |
| 210 | CXCL11 | na | 30753 | -0.213 | -0.2689 | Yes |
| 211 | IL2 | na | 30766 | -0.213 | -0.2637 | Yes |
| 212 | CRLF2 | na | 30784 | -0.214 | -0.2587 | Yes |
| 213 | IL18RAP | na | 30876 | -0.217 | -0.2558 | Yes |
| 214 | CCL20 | na | 30984 | -0.220 | -0.2532 | Yes |
| 215 | LTB | na | 30994 | -0.220 | -0.2478 | Yes |
| 216 | IL25 | na | 30998 | -0.220 | -0.2422 | Yes |
| 217 | TNFRSF4 | na | 31512 | -0.240 | -0.2509 | Yes |
| 218 | IFNA8 | na | 31555 | -0.242 | -0.2459 | Yes |
| 219 | CD40LG | na | 31605 | -0.244 | -0.2410 | Yes |
| 220 | IFNW1 | na | 31920 | -0.257 | -0.2435 | Yes |
| 221 | CCL14 | na | 32172 | -0.268 | -0.2439 | Yes |
| 222 | OSMR | na | 32318 | -0.275 | -0.2410 | Yes |
| 223 | CCL7 | na | 32319 | -0.275 | -0.2339 | Yes |
| 224 | IFNLR1 | na | 32364 | -0.279 | -0.2280 | Yes |
| 225 | TNFRSF1B | na | 32411 | -0.281 | -0.2221 | Yes |
| 226 | PDGFB | na | 32445 | -0.283 | -0.2158 | Yes |
| 227 | IL5 | na | 32563 | -0.292 | -0.2116 | Yes |
| 228 | IFNL1 | na | 32599 | -0.294 | -0.2051 | Yes |
| 229 | INHBA | na | 32642 | -0.297 | -0.1986 | Yes |
| 230 | CSF2RA | na | 32761 | -0.305 | -0.1942 | Yes |
| 231 | INHBE | na | 32770 | -0.306 | -0.1866 | Yes |
| 232 | IL17A | na | 32794 | -0.307 | -0.1793 | Yes |
| 233 | TNFRSF6B | na | 32804 | -0.308 | -0.1716 | Yes |
| 234 | IL10 | na | 32834 | -0.310 | -0.1645 | Yes |
| 235 | CCL22 | na | 32957 | -0.319 | -0.1598 | Yes |
| 236 | TNFRSF9 | na | 33103 | -0.329 | -0.1555 | Yes |
| 237 | CCR9 | na | 33135 | -0.332 | -0.1479 | Yes |
| 238 | GDF5 | na | 33150 | -0.333 | -0.1397 | Yes |
| 239 | CCL21 | na | 33199 | -0.336 | -0.1324 | Yes |
| 240 | IFNA10 | na | 33230 | -0.339 | -0.1246 | Yes |
| 241 | IFNA7 | na | 33254 | -0.341 | -0.1164 | Yes |
| 242 | TGFB3 | na | 33262 | -0.342 | -0.1078 | Yes |
| 243 | CXCL10 | na | 33305 | -0.344 | -0.1002 | Yes |
| 244 | CXCR1 | na | 33444 | -0.355 | -0.0950 | Yes |
| 245 | IL24 | na | 33469 | -0.357 | -0.0865 | Yes |
| 246 | PRLR | na | 33554 | -0.365 | -0.0796 | Yes |
| 247 | IFNK | na | 33679 | -0.376 | -0.0735 | Yes |
| 248 | IL4 | na | 33709 | -0.380 | -0.0645 | Yes |
| 249 | IFNG | na | 33859 | -0.398 | -0.0586 | Yes |
| 250 | CXCR5 | na | 33952 | -0.413 | -0.0506 | Yes |
| 251 | TNFRSF13B | na | 34040 | -0.425 | -0.0422 | Yes |
| 252 | IL22RA2 | na | 34109 | -0.438 | -0.0329 | Yes |
| 253 | IFNA21 | na | 34187 | -0.451 | -0.0235 | Yes |
| 254 | IL11 | na | 34287 | -0.471 | -0.0142 | Yes |
| 255 | ACVRL1 | na | 34306 | -0.475 | -0.0025 | Yes |
| 256 | IL20 | na | 34569 | -0.570 | 0.0046 | Yes |
Table: GSEA details [plain text format]

  

Fig 2: KEGG\_CYTOKINE\_CYTOKINE\_RECEPTOR\_INTERACTION      
 Blue-Pink O' Gram in the Space of the Analyzed GeneSet

  

Fig 3: KEGG\_CYTOKINE\_CYTOKINE\_RECEPTOR\_INTERACTION: Random ES distribution      
 Gene set null distribution of ES for **KEGG\_CYTOKINE\_CYTOKINE\_RECEPTOR\_INTERACTION**
